# Modularity patterns in mammalian domestication: Assessing developmental hypotheses for diversification

**DOI:** 10.1002/evl3.231

**Published:** 2021-06-17

**Authors:** Laura A. B. Wilson, Ana Balcarcel, Madeleine Geiger, Laura Heck, Marcelo R. Sánchez‐Villagra

**Affiliations:** ^1^ School of Biological, Earth and Environmental Sciences University of New South Wales Sydney Australia; ^2^ School of Archaeology and Anthropology The Australian National University Canberra Australia; ^3^ Palaeontological Institute and Museum University of Zurich Zurich Switzerland

**Keywords:** Evolvability, morphological disparity, selection, shape variation, skull, tameness

## Abstract

The neural crest hypothesis posits that selection for tameness resulted in mild alterations to neural crest cells during embryonic development, which directly or indirectly caused the appearance of traits associated with the “domestication syndrome” (DS). Although representing an appealing unitary explanation for the generation of domestic phenotypes, support for this hypothesis from morphological data and for the validity of the DS remains a topic of debate. This study used the frameworks of morphological integration and modularity to assess patterns that concern the embryonic origin of the skull and issues around the neural crest hypothesis. Geometric morphometric landmarks were used to quantify cranial trait interactions between six pairs of wild and domestic mammals, comprising representatives that express between five and 17 of the traits included in the DS, and examples from each of the pathways by which animals entered into relationships with humans. We predicted the presence of neural crest vs mesoderm modular structure to the cranium, and that elements in the neural crest module would show lower magnitudes of integration and higher disparity in domestic forms compared to wild forms. Our findings support modular structuring based on tissue origin (neural crest, mesoderm) modules, along with low module integration magnitudes for neural crest cell derived cranial elements, suggesting differential capacity for evolutionary response among those elements. Covariation between the neural crest and mesoderm modules accounted for major components of shape variation for most domestic/wild pairs. Contra to our predictions, however, we find domesticates share similar integration magnitudes to their wild progenitors, indicating that higher disparity in domesticates is not associated with magnitude changes to integration among either neural crest or mesoderm derived elements. Differences in integration magnitude among neural crest and mesoderm elements across species suggest that developmental evolution preserves a framework that promotes flexibility under the selection regimes of domestication.

**Impact Statement**

Domestication has long captured the attention of evolutionary biologists, owing both to the fascinating array of different forms that it has produced and the similarity in features among these forms. The neural crest hypothesis represents an appealing explanation for how domestic forms have been generated by offering a simple, unitary underlying cause for traits considered part of the “domestication syndrome” (DS). Under this hypothesis, selection on tameness has resulted in genetic changes affecting development of neural crest cells, thereby causing features of the DS. Although the neural crest hypothesis has gained traction, support from morphological data and for the validity of the DS remains a topic of debate. In this paper, we use the quantitative frameworks of modularity and integration to test a series of morphological‐based predictions relating to domestication and the neural crest hypothesis. We sampled a broad range of domestic/wild comparisons, including representatives from each of the three pathways (commensal, directed, prey) that are recognised for human‐animal interactions. We predicted that if the neural crest was responsible for changes associated with domestication, bones of the skull derived from neural crest cells would show more variation than those derived from the mesoderm, when comparing wild and domestic forms. Overall, our results indicate greater variation in skull shape among domesticated versus wild mammals, and that skull bones derived from the neural crest differ in their variation and interactions. Contra to our predictions, we find that greater variation in domesticates is not associated with magnitude changes to trait interactions among either neural crest or mesoderm derived bones. We advance the understanding of trait interactions during domestication, showing that the generation of disparity appears to proceed by co‐opting underlying trait relationships.

The domestication process involves different degrees of human‐animal association (Vigne 2011; Zeder 2012). After initial interactions, prolonged and directed relationships can originate via different pathways. Through selective breeding (Van Grouw 2018), a desired outcome is obtained, for example, meat production, transport of goods, ornamentation, and companionship. Domestication in all its forms has brought about an array of features in domestic animals that are not shared by their wild counterparts (Darwin 1868; Herre and Röhrs 1990).

Darwin (1868) first identified features that were common among distantly related domesticated mammals. These traits have more recently been described as the “domestication syndrome” (DS) (see Lord et al. 2020a for historical overview). Besides behavioural features, the DS includes morphological features such as floppy and reduced ears, smaller teeth, smaller cranial capacity, loss of pigmentation, curly tail and a shorter muzzle (Herre and Röhrs 1990; Price 2002) (Fig. 1A). The occurrence of these traits is variable across species (Darwin 1868; Wilkins et al. 2014), an observation that has been highlighted recently (Sánchez‐Villagra et al. 2016; Wilkins 2017, 2020), along with a critical examination of the concept (Lord et al. 2020a).

**Figure 1:**
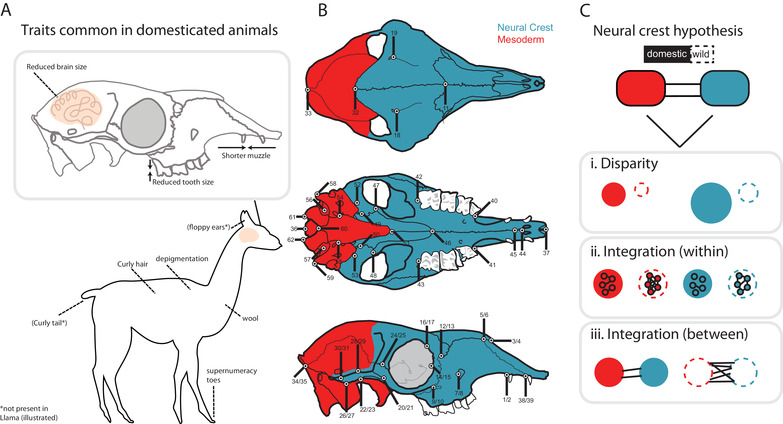
Schematic illustration of the hypothesis framework implemented in this study. (A) Domesticated animals share, to a varying degree, a suite of traits. These traits are hypothesized to have been caused by disruption in neural crest development (the neural crest hypothesis; Wilkins et al. 2014). (B) The neural crest is responsible for the patterning of some cranial bones, studied here using geometric morphometric landmarks collected on domestic/wild mammals, as seen in dorsal (top), ventral (middle), and lateral (bottom) views. Landmarks were subset into two modules, Neural Crest (NC, blue) and Mesoderm (MD, red), based on tissue origin of cranial elements, after Mischina and Snider (2014). (C) The neural crest hypothesis was evaluated using measures of modularity and integration, under the following predictions: i) module disparity will be greater for domestic versus wild forms, and module disparity for the NC module will be greater than that for the MD module, ii) within‐module integration magnitudes will be lower for domestic forms versus wild forms, and integration magnitudes will be lower for the NC module compared to the MD module, and iii) measures of between module integration will be lower for domestic forms compared to wild forms. Cranial illustrations modified after Balcarcel et al. (2021). Not all landmarks illustrated could be recorded for all species due to species‐specific cranial morphology. Landmark descriptions are provided in Table S1.

The initial phases of domestication involved selection for tameness and docility, sparked in some cases by the attraction of animals to the human niche and the advantages it conferred (Zeder 2012; Larson and Fuller 2014). Selection for such behavioral features is reflected in changes to the sympathetic nervous system threat response (“fight‐or‐flight”), and has been proposed to lead to neural crest cell deficiencies during development (Wilkins et al. 2014). Specific to vertebrates, the neural crest is a population of pluripotent cells that undergo a ventral migration through the cranium and trunk during embryogenesis (Hall 1999; Noden and Schneider 2006). Besides being responsible for the development of bones (Fig. 1B), cartilage and connective tissue, neural crest cells are also involved in development of the adrenal glands, which produce adrenocorticotropic hormones that initiate the “fight‐or‐flight” response. The neural crest hypothesis proposes that changes in neural crest cell functioning during early development, particularly aberrant migration, have produced the suite of DS traits as a by‐product (Wilkins et al. 2014; Wilkins 2017). Thus, there is a developmental basis for the DS, involving several small changes across many genes that influence neural crest cell development (Wilkins et al. 2014), with no single genetic change being necessary or sufficient for domestication (Carneiro et al. 2014).

The neural crest hypothesis has gained traction. Features of the genomes of horses (Schubert et al. 2014; Librado et al. 2017), cats (Montague et al. 2014), camels (Fitak et al. 2020), rabbits (Carneiro et al. 2014), and dogs (Axelsson et al. 2013; Pendleton et al. 2018) have been interpreted as supporting the hypothesis because domestic breeds show mutations in neural crest cell genes that are absent in their progenitors (Wilkins 2017). On the other hand, the significance of genomic studies in supporting the neural crest hypothesis has been questioned (Johnsson et al. 2021). Exploration of morphological trait variation under this hypothesis is much needed (Sánchez‐Villagra et al. 2016; Sánchez‐Villagra and Van Schaik 2019).

The validity of the DS, and the conditions under which its expectations are met, are subject of controversy (Lord et al. 2020a,b,c; Wright et al. 2020; Zeder 2020). There are no universal patterns of behavioral (Wheat et al. 2019, 2020) or morphological (Sánchez‐Villagra et al. 2017) features in domestic mammals (Lord et al. 2020a). However, the role of the neural crest in morphological differentiation is uncontested and fundamental in the generation of morphological diversity under any selection regime (Schneider 2005, 2018). As such, the quantification of patterns of variation, in light of the embryonic tissues that generate the skull, should provide information on multiple modes of evolution (Rehkämper et al. 2008; Parsons et al. 2020). The adaptive response of individual species to selection under domestication may align with macroevolutionary patterns of diversification (Young et al. 2017), thereby yielding insight into the capability of shared developmental systems to generate phenotypic variation.

Morphological studies have uncovered a lack of commonality in ontogenetic processes among domestic forms (Sánchez‐Villagra et al. 2017; Wilson 2018), highlighting the importance of investigating and comparing diverse species. This study focuses on one of the most prominent aspects of domestication – the morphological variation that it has produced in the mammalian cranium – and adopts the frameworks of modularity and integration to test morphological predictions associated with the neural crest hypothesis. Integration is the covariation or correlation among traits, whereas modularity describes subsets of tightly integrated traits (modules) that are weakly connected to other subsets (Olson & Miller 1958; Wagner & Altenberg 1996; Klingenberg et al. 2004). The two concepts are inter‐twined, measuring the pattern and magnitude of organization in anatomical traits that primarily result from shared developmental, functional, or genetic relationships (Wagner et al. 2007; Klingenberg 2008). Modularity has been shown to facilitate evolvability, or to promote the evolution of complexity by enabling different subsets (modules) to evolve quasi‐independently (Goswami et al. 2014; Larouche et al. 2018), whereas high integration magnitudes have been considered to result in reduced capacity for evolutionary response, whereby changes in one trait may exact a negative effect on the function of closely integrated traits (Melo et al. 2016; Felice et al. 2018). Within‐module integration levels have also been linked to the generation of disparity: lower integration within modules is linked to higher magnitudes of cranial disparity in mammals (Goswami and Polly 2010a) and archosaurs (Felice et al. 2018; Lee et al. 2020). Thus, the architecture of trait interactions may affect an organism's response to selection, directing lineage diversification along favored pathways (Shirai and Marroig 2010; Melo et al. 2016), and making quantification of these interactions necessary to understanding morphological evolution under different selection regimes.

The role of modularity and integration in the generation of cranial disparity through domestication has been examined for the dog (Drake and Klingenberg 2010; Parr et al. 2016; Curth et al. 2017; Machado et al. 2018; Selba et al. 2020; Brassard et al. 2020). In general, modularity patterns are conserved between dogs and wolves, echoing the results of broader comparisons across mammals (Porto et al. 2009). In contrast, dogs and wolves reportedly differ in cranial integration magnitudes (Parr et al. 2016; Curth et al. 2017; Machado et al. 2018), a result that has been similarly recovered across broader taxonomic sampling, including in other domesticates (Young et al. 2017; Stange et al. 2018). This has been proposed to signal the importance of magnitude changes against a backdrop of stasis in modularity patterns (Marroig et al. 2009). Conservatism in patterns of modularity have been attributed to stabilizing selection acting on the effects of developmental processes (Hallgrimsson et al. 2007). However, when developmental mechanisms are disrupted or altered, for example through mutations, trait interactions have been shown to change (Jamniczky & Hallgrimsson 2009). Deficits in neural crest cell functioning, as predicted under the neural crest hypothesis, may therefore be reflected in measurable changes in cranial trait interactions.

We begin with the basic assumption that if neural crest cells are central to the DS, then their developmental products, here cranial elements, should show greater change in domestic compared to wild forms. This change is assumed to be supported through lower magnitudes of integration, whereby weaker trait interactions manifest through relatively relaxed selection pressures associated with incursion of domesticates into a human‐occupied environment. We first test the assumption that one may uncover relatively strong connections between cranial elements that derive from the neural crest (NC), expecting that they form a module relative to other cranial elements, which derive from the mesoderm (MD) (Fig 1; Mishiner & Snider 2014; Koyabu et al. 2014). We test this across six pairs of domestic/wild mammals, predicting support for NC and MD modular structure of the cranium in all forms. Using this modular framework, we then test several hypotheses on how the NC module may respond quasi‐independently, showing greater variation in domestic forms: (H1) module disparity (both NC and MD) will be greater for domestic versus wild forms, as seen in other domestic mammals, and disparity in the NC module will be greater than that for the MD module, reflecting variation potentially associated with changes to neural crest cell functioning under domestication *sensu stricto* and selective breeding (Fig. 1C, part i); (H2) within‐module integration magnitudes will be lower for domestic versus wild forms, following evidence that low integration magnitudes facilitate response to selection and promote disparity, and moreover, integration magnitudes will be lower for the NC module compared to the MD module (Fig. 1C, part ii), in line with expectations of a role for integration magnitude differences in domestication; (H3) between‐module integration will be lower for domestic compared to wild forms, consistent with the preserved developmental pathways in wild forms and contrasting with fewer between‐module connections in domestic forms, which will allow responses to directional selection along independent pathways (Fig. 1C, part iii).

## Methods

### SAMPLE COMPOSITION

We compiled data for 539 adult individuals, representing data collected from six domestic/wild mammalian pairs (Supplementary Data 1): Goat/Bezoar (*Capra hircus*/*Capra aegagrus*) (N = 41/22, total = 63); Dog/Wolf (*Canis lupus familiaris*/*Canis lupus*) (N = 45/25, total = 70); Pig/Wild Boar (*Sus scrofa domestica*/*Sus scrofa scrofa*) (N = 22/28, total = 50); Horse/Przewalski's Horse (*Equus ferus caballus*/*Equus ferus przewalski*) (N = 133/83, total = 216); Llama/Guanaco (*Lama glama*/*Lama guanicoe*) (N = 18/86, total = 104) and Alpaca/Vicuña (*Vicugna pacos*/*Vicugna vicugna*) (N = 14/21, total = 35).

### GEOMETRIC MORPHOMETRIC DATA COLLECTION

Three‐dimensional (3D) cranial landmark data were digitized using a Microscribe digitizer (MLX, Revware, Inc., USA) and Microscribe Utility Software (MUS: v.7.0.1.1). Left and right sides were captured. A total of 62 cranial landmarks were digitized for Pig/Wild Boar, Horse/Przewalski's Horse, Llama/Guanaco, and Alpaca/Vicuña. Data for each species were sampled by one author. Two landmarks, recording the posterior tip of the “canine” alveolus (first tooth anterior to diastema) (LM#1 and #2), were absent for the Goat/Bezoar (n = 60) due to their absence in this clade (Table S1). Sampling was supplemented with cranial landmarks for Dog/Wolf, consisting of a subset (n = 26) of the 62 landmarks, taken from Geiger et al. (2017).

This study focuses on a homologous set of landmarks and their assignment to modules. Comparison across modules was permitted by accounting for differences in intra‐module landmark number, following previous approaches for modularity analysis based on modules containing unequal landmark numbers (e.g., Goswami and Polly 2010b; Bardua et al. 2019). Data sets were not pooled for global sample estimates of modularity, nor for the ordination, assessment, or description of domestic/wild differences in cranial shape, which have been the subject of study elsewhere (Parr et al. 2016; Geiger et al. 2017; Heck et al. 2018; Balcarcel et al. 2021).

### DATA ANALYSES

#### Generalized procrustes analysis

Raw landmark matrices (N = 6) were created for each domestic/wild pair. Generalized Procrustes Analysis (GPA) (Rohlf and Slice 1990) was performed on each domestic/wild pair using the function gpagen in the R package geomorph version 3.1.3 (Adams et al. 2019) and the symmetric component of shape was extracted using the function bilat.symmetry. Landmarks were not pooled for all species because the focus of this study was on intraspecific comparison of modularity and integration and not interspecific cranial shape differences, which would swamp comparisons of intra‐module variation within domestic/wild pairs. We follow Baab (2013) and adopt a simultaneous Procrustes fit approach, followed by subsetting of landmarks into modules (see Supporting Information S1 for details).

#### Allometry

Procrustes ANOVA was performed using the procD.lm function in geomorph version 3.1.3 (Adams et al. 2019), and residuals were extracted for each species. The pairwise function in RRPP version 0.4.3 (Collyer and Adams 2018) was used to assess differences between domestic/wild forms and between module partitions (see Supporting Information S2). Linear models were assessed for statistical significance using residual randomization with 1000 permutations (Collyer et al. 2015). Both uncorrected shape data and allometry‐corrected data (residuals) were analyzed; size changes hold evolutionary importance in the study of domestication and a significant relationship between shape and size was uncovered for each domestic/wild pair (Table S2). Cranial centroid sizes were compared with T‐tests for each domestic/wild pair using the R base function t.test().

For each pair, variance differences between wild and domestic groups were assessed using both the allometry‐corrected and uncorrected shape data, implemented with the morphol.disparity function in R package geomorph version 3.1.3 (Adams et al. 2019).

#### Modularity and integration tests, whole cranium

Cranial landmarks were a priori assigned to a neural crest (NC) or mesoderm (MD) module (Text S2). To assess support for our hypothesis of NC‐MD modularity, we calculated the covariance ratio (CR) (Adams 2016) using the modularity.test function in the R package geomorph version 3.1.3 (Adams et al. 2019) for each domestic/wild pair (see Supporting Information S2).

For each domestic/wild pair, the strength of covariation (integration) between the Procrustes coordinates in the NC and MD modules was assessed using a two‐block partial least‐squares (PLS) analysis with geomorph function integration.test (Collyer et al. 2015; Adams and Collyer 2016), referred to as a singular warp analysis for landmark data (Bookstein et al. 2003; Rohlf and Corti 2000). This test was performed on the landmark data and residuals. The outputted mean pairwise correlation between the NC and MD partitions was considered significant if greater than the distribution of values obtained by randomly permuting individuals in one partition relative to the other (1000 permutations) (Adams and Collyer 2019). To assess differences in covariation magnitude between NC and MD modules across domestic/wild pairs, effect sizes were extracted and compared between the PLS analyses for all pairs (excluding Dog/Wolf with fewer landmarks) using the compare.pls function in geomorph. The PLS results were used to assess the extent to which the main axis of shape variation in the domestic/wild pairs corresponds with covariation between the NC and MD modules (see Supporting Information S3).

#### Module disparity and integration hypotheses

##### Hypothesis H1 – Higher magnitudes of module disparity for domestic forms and the NC

For each domestic/wild matrix of allometry‐corrected Procrustes landmarks, the magnitude of variation (disparity) in the landmarks assigned to the NC and MD modules was quantified using the morphol.disparity function in geomorph (Fig. 1C, part i). Morphol.disparity estimates disparity using Procrustes variance, the trace of the covariance matrix divided by sample size. Residual randomization permutation (1000 permutations) was used to assess differences in disparity values for wild and domestic forms within each module. Disparity values for each module were divided by the number of landmarks in the module to account for unequal landmarks and enable comparison between modules (see Heck et al. 2018; Bardua et al. 2019 for similar approaches).

##### Hypothesis H2 – Lower magnitudes of within‐module integration for domestic forms and the NC

Within‐module integration was calculated for NC and MD modules using relative eigenvalue standard deviation (Eigenvalue dispersion) (Pavlicev et al. 2009) (Fig. 1C part ii). Eigenvalue dispersion values were calculated from a singular value decomposition of the correlation matrix for each module, to provide a trait‐independent measure of integration (Goswami and Polly 2010b: equation 7). Eigenvalue dispersion values range between 0 and 1, with larger values reflecting a greater proportion of shape variation concentrated within a small number of eigenvectors, indicating a greater degree of integration (Pavlicev et al. 2009).

##### Hypothesis H3 – Lower magnitudes of between‐module integration for domestic forms

To examine differences in between‐module integration for domestic versus wild forms (Fig. 1C part iii), the strength of covariation (integration) between the Procrustes coordinates in the NC and MD modules was assessed using a two‐block partial least‐squares (PLS) analysis implemented in geomorph with the function integration.test (Collyer et al. 2015; Adams and Collyer 2016). This test was performed separately on the landmark data, and on residuals, for each domestic and wild form.

To assess whether there was a relationship between morphological disparity and integration for the NC and MD modules, we performed two sets of regressions using R base function lm(). Morphological disparity was regressed against within‐module integration (Eigenvalue dispersion) values and against between‐module integration (z‐scores extracted from the PLS analyses) effect size values.

All analyses were performed in the R version 3.6.1. environment (R Core Team 2019).

## Results

### ALLOMETRY AND VARIANCE

The regression of Procrustes shape data onto log Centroid size revealed a significant effect of allometry for all domestic/wild pairs (Table S2). Separate pairwise comparisons of allometric vectors for domestic/wild pairs and for module partitions (Table S3) did not reveal significant differences. The amount of shape variance explained by size ranged between 6 % (Llama/Guanaco) and 23 % (Pig/Wild Boar) across all pairs (Table S2). Cranial centroid sizes differed significantly between wild and domestic forms for most pairs, the wild group being larger in half of comparisons (Table S4, Figure S1)

Procrustes variance values for allometry‐corrected (residuals) and uncorrected shape data indicated that for most species, domestic forms have significantly greater dispersion than wild forms (Table S5, Figure S2).

### MODULARITY AND INTEGRATION TESTS, WHOLE CRANIUM

Modularity analyses revealed significant support for the a priori division of cranial landmarks into neural crest (NC) and mesoderm (MD) modules. In all cases, CR values were lower than 1, indicating greater covariation within‐modules than between‐modules, supporting a modular structure for the cranial landmarks. CR values ranged between 0.77 (Horse/ Przewalski's Horse and Dog/Wolf) and 0.89 (Pig/Wild Boar) for the residuals (Table S6A) (average CR = 0.81). A similar result was recovered for the uncorrected landmark data (Table S6B) (average CR = 0.83), with the exception that the CR value for Goat/Bezoar was lower and significant for the uncorrected data but not for residuals.

Correlations between Principal Component 1 (PC1) scores extracted from Principal Component Analysis (Tables S7‐S12) and Partial Least Squares axis 1 (PLS1) scores revealed a significant relationship for four out of six domestic/wild pairs, suggesting some alignment between the main direction of shape variation and between‐module integration for the majority of species (Table S13). However, correlations were highly variable for both the allometry‐corrected and uncorrected data, with values ranging between 0.03 (Alpaca/Vicuña, not significant) and 0.97 (Pig/Wild Boar) (Table S13). Allometry‐corrected data yielded lower correlation values (range 0.03‐0.63) for all domestic/wild pairs in comparison to correlations on uncorrected shape data, indicating that size‐correlated shape variation is more closely aligned with covariation between NC and MD modules and supporting the role of size as an integration factor (see Porto et al. 2013). The difference in correlation values between allometry‐corrected and uncorrected shape data was most pronounced for Pig/Wild Boar (0.47 compared to 0.97 respectively) (Table S13), in line with the greater magnitude of variance explained by size for this pair (Table S2).

Pairwise comparisons (z, effect size) of PLS analyses for all pairs, excluding Dog/Wolf with fewer landmarks, showed that levels of integration between the NC and MD modules were highest for Horse/ Przewalski's Horse (z = 15.63 uncorrected shape data, z = 13.78 allometry‐corrected data) and Pig/Wild Boar (z = 9.77 uncorrected shape data, z = 7.61 allometry‐corrected data) (Table S14). Effect sizes differed significantly between most domestic/wild pairs for uncorrected shape data, except for Alpaca/Vicuña and Goat/Bezoar (Table S15).

### WITHIN MODULE DISPARITY AND INTEGRATION

#### Hypothesis H1 – Higher magnitudes of module disparity for domestic forms and the NC

**Figure 2:**
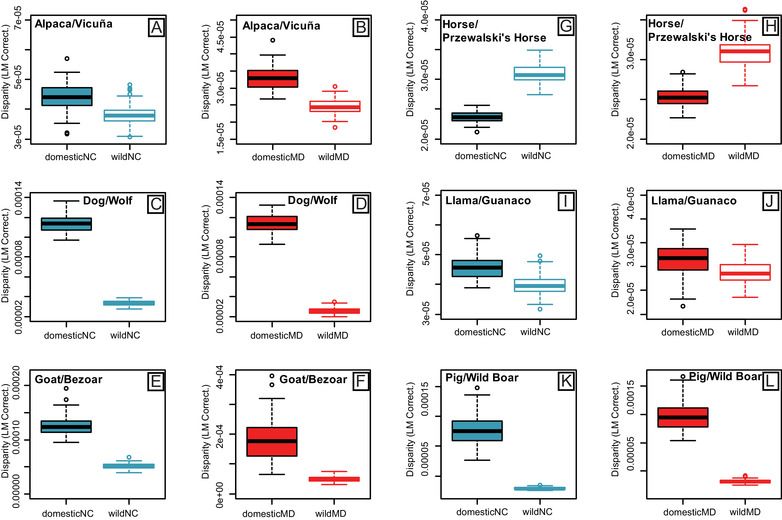
Boxplots of morphological disparity for cranial landmarks assigned to the Neural Crest (NC) (blue) and Mesoderm (MD) (red) modules, displaying values for six domestic/wild mammal pairs (A‐B, C‐D, E‐F, G‐H, I‐J, K‐L). Disparity was measured as Procrustes Variance and was corrected by the number of landmarks in each module, to enable direct comparison. Disparity was calculated on Procrustes superimposed landmarks (graphed) and residuals (Table S16).

**Figure 3:**
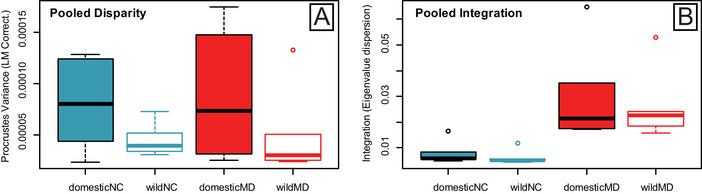
Boxplots of (A) morphological disparity and (B) within‐module integration pooled for cranial landmarks assigned to the Neural Crest (NC) (blue) and Mesoderm (MD) (red) modules. Values are pooled for six wild and domestic mammal pairs. Procrustes Variance and Eigenvalue dispersion values were corrected for the number of landmarks in each module, to enable direct comparison. Disparity and Integration values were calculated using Procrustes superimposed landmarks (graphed) and residuals (Table S16 and Table S18).

Module disparity values for allometry‐corrected and uncorrected shape data were higher for domestic forms compared to wild forms for both the NC and MD modules (Fig. 2), with the exception of Horse/Przewalski's Horse (Table S16) (Fig. 2G‐H). Significant differences between wild and domestic disparity values were uncovered for five of six pairs for the MD module, and for four of six pairs for the NC module (Table S16, allometry‐corrected data). Llama/Guanaco did not show significant differences in disparity for either module (Fig. 2I‐J). Variance in morphological disparity was higher for domestic compared to wild forms across both modules, though confidence intervals overlapped (F_22,1_ = 4.206, *P* = 0.052; Fig 3A). Across all pairs, NC module disparity was not higher than MD module disparity for allometry‐corrected (*P* = 0.96) and uncorrected (*P* = 0.36) shape data.

#### Hypothesis H2 – Lower magnitudes of within‐module integration for domestic forms and the NC

Within‐module integration values, measured using relative eigenvalue standard deviation, were low for both NC and MD modules and across all pairs, indicating low magnitudes of integration (Fig. 3B). Comparatively, integration values were higher for the mesoderm module (average all pairs, 0.028) relative to the neural crest module (average all pairs, 0.007) (*t* = −4.515, *P* = 0.0007) (Fig. 3B). Thus, cross module (i.e., NC wild vs. MD wild and NC domestic vs. MD domestic) comparisons were significant for both wild (*t* = −3.535, *P* = 0.015) and domestic (*t* = −2.796, *P* = 0.034) forms (Table S17). However, across form comparisons (i.e., NC wild vs. NC domestic and MD domestic vs. MD domestic) were not significant. Compared to wild forms, domestic forms had slightly higher integration variance across species, although this was not significant (F_22,1_ = 0.158, *P* = 0.694).

#### Hypothesis H3 – Lower magnitudes of between‐module integration for domestic forms

Between‐module integration was highly similar for domestic and wild forms, ranging between 0.79 and 0.92 for domesticates and between 0.77 and 0.93 for wild forms (Supplementary Table S18), using residuals. Values for uncorrected data (average 0.84) were similar to those for allometry‐corrected data (average 0.85). Differences between wild and domestic integration values were compared with a *t*‐test that did not reveal significant differences (*P* = 0.89).

**Figure 4:**
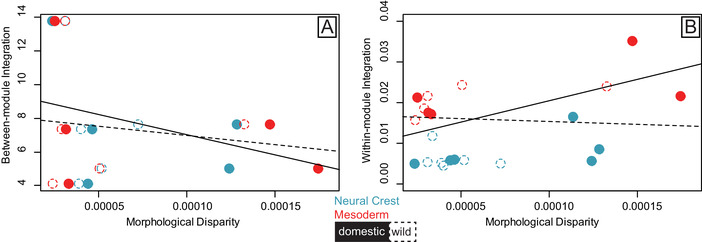
Scatterplots of the relationship between (A) morphological disparity (Procrustes Variance) and between‐module integration (z‐scores for between module PLS), and (B) morphological disparity (Procrustes Variance) and within‐module integration (Eigenvalue dispersion). Values calculated from cranial landmarks assigned to the Neural Crest (NC) (blue) and Mesoderm (MD) (red) modules for six domestic/wild pairs of mammals. Ordinary least squares regression lines show fit for the pooled (MD and NC) relationships for domestic (solid line) and wild (dashed line) forms. Filled ellipses are the domestic form and open ellipses are the wild form. Disparity and Integration values were calculated using Procrustes superimposed landmarks (graphed) and allometry‐corrected residuals (Table S16 and Table S18).

The relationship between morphological disparity and between‐module integration (Fig. 4A) (integration ∼ disparity*domestic/wild) was not significant (*P* = 0.265), with similar slopes for both wild and domestic forms, largely being impacted by the high effect size (z‐score) for Horse/Prezwalski's Horse (Supplementary Table S14). Similarly, the relationship between morphological disparity and within‐module integration (Eigenvalue dispersion) (Fig. 4B) was not significant (*P* = 0.243) and slopes were similar for both wild and domestic forms (F_22,1_ = 0.537, *P* = 0.577). Taken together, we find that integration magnitudes (within or between modules) do not constrain morphological disparity in either wild or domestic forms.

## Discussion

Our results support a modular structuring of the cranium based on neural crest (NC) and mesoderm (MD) modules and differences in integration magnitude. The latter are consistent with foundational studies on the evolutionary importance of trait interactions (Marroig & Cheverud 2005; Porto et al. 2009; Marroig et al. 2009) and suggest unequal capacity for evolutionary response between NC‐ and MD‐derived elements. Covariation between NC and MD modules was found to account for major components of shape variation for most domestic/wild pairs. Support for an NC‐MD division of the cranium is consistent with other studies testing rostrum‐braincase module division in adult forms (Drake and Klingenberg 2010; Martínez‐Abadías et al. 2012; Singh et al. 2012; Cardini and Polly 2013; Cardini et al. 2015; Curth et al. 2017; Selba et al. 2020).

After confirming NC‐MD modularity, our downstream hypotheses targeted two sets of comparisons: those between wild and domestic forms and those between NC and MD modules. Across species, we anticipated that domestic forms would have higher module disparity and lower integration magnitudes (within‐ and between‐modules), which is consistent with low integration magnitudes potentially facilitating the generation of disparity under domestication (Curth et al. 2017). For the second set of comparisons, NC modules were predicted to display higher disparity and lower integration than MD modules, pointing towards an influence of regulatory changes to neural crest cells expected under the neural crest hypothesis (Wilkins et al. 2014), and evidence that trait interactions may change when development is disrupted (Jamnickzy and Hallgrimsson 2009). Module disparity was higher for domesticates compared to wild forms but, contra to our predictions, was not significantly higher for NC compared to MD modules. Within‐module integration magnitudes were lower for NC compared to MD modules as predicted, whereas within‐ and between‐module integration was not lower for domesticates compared to wild forms. Altogether, this indicates that NC cranial modules are less integrated for all forms, and that domesticates have a higher disparity in the cranium. It was not possible to connect the low integration magnitudes of the NC module with higher module disparity, in the absence of a significant relationship between them (Fig. 4).

The results suggest that integration magnitude changes are prevalent, occurring across modules (NC vs MD) and between species but not within domestic/wild pairs. In the context of trait interactions, the evolutionary history of the mammalian skull has been suggested to be one of magnitude rather than mode changes (Marroig et al. 2009; Machado et al. 2018). Therefore magnitude differences between species are not surprising. These changes have been interpreted as central to the evolutionary flexibility of form that has been realized across mammals and different time scales. Our findings suggest that selection associated with tameness and selective breeding has not led to appreciable changes in integration magnitudes for domesticates relative to their wild progenitors. A similar result was recovered with a narrower sampling, focused on comparing the rostrum and braincase regions in wolves and dogs (Curth et al. 2017). As such, variation under domestication has been generated with a prevailing modular structuring of the cranium. However, integration magnitude changes do not appear to be connected to the diversification of shape among domesticates sampled herein. We find that shape variation between domesticated and wild forms is explained by covariation between the NC and MD cranial modules, which also suggests that increased disparity in domesticates is achieved through the co‐opting of a common set of trait interrelations. From an evolutionary perspective, highly integrated modules in the skull are hypothesized to be under intense selection to preserve the many, complex functions of the head (Lieberman 2011). The combination of diverse functions, high modularity, and strong integration is hypothesized to promote evolvability in the head (Lieberman 2011). The assumed reductions in selection pressure associated with incursion into the human niche (e.g., increased food availability, reduced predation) may not have been significant enough to alter these connections. There is some theoretical (Goswami et al. 2014) and empirical (Navalon et al. 2020) evidence that evolution along restricted paths may not negatively impact the generation of disparity.

Lower integration magnitudes for the NC module in both domestic and wild forms were partially consistent with our predictions that connections among these traits would show magnitude differences compared to the MD module. However, differences were not detected between domestic and wild forms nor were lower integration magnitudes linked to higher disparity among traits in that module. This reflects decoupled variation for the two measures; integration magnitude varied by module whereas module disparity varied by domestic/wild form (Fig. 2). Therefore, our predictions about the role of neural crest cell derived traits in generating disparity under domestication are not unanimously supported. We instead consider that changes in cranial morphology among domesticates were accompanied by low integration magnitudes in the facial region in general, with these having persisted from the wild type.

We here present an initial step forward, examining trends across several cases of mammalian domestication to assess commonalities and differences within a trait covariance framework, and thus an approach that addresses the challenges in testing assumptions of the neural crest hypothesis using morphological data (Sánchez‐Villagra et al. 2016). More complex scenarios, relating modulation of neural crest variation and integration magnitudes, may exist, especially those concerning the alignment of selection and body size change under domestication (Porto et al. 2013; Wilson 2018). We also acknowledge our results may be impacted by sample size, stage of domestication, and differences among domesticates in the length of time separating sibling lines. In particular, the ancestry of Przewalski's Horse has recently been revisited and genetic data indicate it may have some domestic heritage (Gaunitz et al. 2018). Modern wild populations may differ between geographic locations and in their presentation of traits compared to ancestral pre‐domesticated wild populations (Lord et al. 2020c), which are not preserved or available for study.

Debate has recently arisen surrounding the complex nature of the DS, and how many changes we may expect to count as evidence in favor of its validity (Lord et al. 2020a, 2020b; Zeder 2020). A review of all traits attributed to the DS indicated that changes to soft tissue features (e.g., curled tail, depigmentation, floppy ears) were more strongly supported by published data than skeletal features (Lord et al. 2020a; Kistner et al. 2021). Since we show that module disparity and integration are not correlated across domesticates, testing the contribution of evolutionary rates is warranted. Higher evolutionary rates may enable higher disparity in domesticates under conditions of both low and high integration magnitudes. That is, rate shifts may be independent of integration, thereby offering a pathway to diversification under divergent selection regimes or different temporal scales (e.g. timelines associated with the different pathways into domestication). Higher rates have been hypothesized under conditions of high disparity and integration (Felice et al. 2018). Notably, evolutionary rates in domesticates have been shown to be both faster than (Castaglione et al. 2021), and the same as (Geiger et al. 2018), wild mammals.

The absence of strong support for our hypotheses does not refute the potential validity of the neural crest hypothesis per se, but we conclude that it lacks agency based on data on morphological integration and modularity of the adult skull. However, we have illuminated the understanding of trait interactions during domestication, showing that the generation of disparity appears to proceed by co‐opting underlying trait relationships. By implication, differences in integration magnitude among neural crest and mesoderm elements across species suggest that developmental evolution preserves a framework that promotes flexibility under the selection regimes of domestication.

## AUTHOR CONTRIBUTIONS

L.A.B.W. conceived of the study, analyzed the data, and drafted the manuscript; A.B., M.G., and L.H. collected data; A.B., M.G., and M.R.S.‐V. contributed to study design and conception and edited drafts of the manuscript; all authors approved the final version of the manuscript.

## DATA ARCHIVING

All raw data are provided in the supporting files.

## CONFLICT OF INTEREST

The authors declare no conflict of interest.

## Supporting information

Supporting Information

**Table S1**. Description of three dimensional (3D) geometric morphometric landmarks (LM) collected on mammalian crania for wild/domestic pairs in this study and their assignment to neural crest (unshaded) and mesoderm (shaded grey) modules.
**Table S2**. Results of Procrustes ANOVA (shape ∼ size), test statistics based on Residual Randomization (1000 permutations).
**Table S3**. Pairwise statistics for comparisons of LS mean vector correlations between wild/domestic forms and between landmarks in the Neural Crest (NC) and Mesoderm (MD) modules based on residual randomization 1000 permutations. The null hypothesis is that the angle between vectors = 0.
**Table S4**. Welch's two sample T‐test results for comparisons of log centroid size between wild and domestic forms.
**Table S5**. Comparisons of morphological disparity between groups (wild/domestic) for (A) allometry‐corrected and (B) uncorrected shape data (residuals), providing Procrustes Variance values for each group and a P‐value associated with pairwise differences in variances, based on resampling (1000 permutations).
**Table S6**. Results of modularity and integration tests using allometry‐corrected shape data (residuals) (A) and uncorrected shape data (B), using two a priori defined modules for the cranium: the neural crest (NC) and mesoderm (MD).
**Table S7**. Summary of the Principal Component (PC) axes extracted from Principal Component Analysis (PCA) of cranial landmark data for *Canis lupus*/*Canis lupus familiaris*, detailing 95% of sample variation.
**Table S8**. Summary of the Principal Component (PC) axes extracted from Principal Component Analysis (PCA) of cranial landmark data for *Capra aegagrus/Capra hircus* detailing 95% of sample variation.
**Table S9**. Summary of the Principal Component (PC) axes extracted from Principal Component Analysis (PCA) of cranial landmark data for *Equus ferus przewalskii/ Equus ferus caballus* detailing 95% of sample variation.
**Table S10**. Summary of the Principal Component (PC) axes extracted from Principal Component Analysis (PCA) of cranial landmark data for *Lama guanicoe/Lama glama* detailing 95% of sample variation.
**Table S11**. Summary of the Principal Component (PC) axes extracted from Principal Component Analysis (PCA) of cranial landmark data for *Sus scrofa scrofa/Sus scrofa domestica* detailing 95% of sample variation.
**Table S12**. Summary of the Principal Component (PC) axes extracted from Principal Component Analysis (PCA) of cranial landmark data for *Vicugna vicugna/Lama pacos* detailing 95% of sample variation.
**Table S13**. Results of vector correlations between Principal Component 1 (PC1) and Partial Least Squares axis 1 (PLS1) for landmark data and allometry‐corrected landmark data.
**Table S14**. Effect sizes (z) extracted from pairwise comparisons of Partial Least Squares (PLS) analyses for landmark data and allometry‐corrected landmark data.
**Table S15**. Pairwise comparison of effect size (z) (raw values presented in Table S12) using two‐sample z‐tests of PLS analyses, showing P values for allometry‐corrected data (below diagonal) and uncorrected data (above diagonal).
**Table S16**. Morphological disparity values, measuring within‐module disparity for the Neural Crest (NC) and Mesoderm (MD) modules, for wild and domestic forms. Values calculated for A) Allometry‐corrected and B) Uncorrected shape data. All disparity values corrected for unequal number of landmarks within each module (disparity/N landmarks).
**Table S17**. Eigenvalue dispersion values, measuring integration magnitude within the Neural Crest (NC) and Mesoderm (MD) modules, for wild and domestic forms. Integration values are corrected for unequal number of landmarks within each module (relative standard deviation, see Pavlicev et al. 2009).
**Table S18**. Between‐module integration values for wild and domestic forms, assessing integration between the Neural Crest (NC) and Mesoderm (MD) modules.
**Figure S1**. Boxplots of log cranial centroid size for wild (yellow) and domestic (blue) mammal pairs.
**Figure S2**. Ordinations of uncorrected landmark data, for wild (yellow) and domestic (blue) forms.
