# Supplementary material for: Modularity patterns in mammalian domestication: Assessing developmental hypotheses for diversification
Source: Evol Lett. 2021 Jun 17;5(4):385–96. doi: 10.1002/evl3.231 (PMC8327948; doi:10.1002/evl3.231)
Supplement: Supplementary file 2 — Table S1. Description of three dimensional (3D) geometric morphometric landmarks (LM) collected on mammalian crania for wild/domestic pairs in this study and their assignment to neural crest (unshaded) and mesoderm (shaded grey) modules. Table S2. Results of Procrustes ANOVA (shape ∼ size), test statistics based on Residual Randomization (1000 permutations). Table S3. Pairwise statistics for comparisons of LS mean vector correlations between wild/domestic forms and between landmarks in the Neural Crest (NC) and Mesoderm (MD) modules based on residual randomization 1000 permutations. The null hypothesis is that the angle between vectors = 0. Table S4. Welch's two sample T‐test results for comparisons of log centroid size between wild and domestic forms. Table S5. Comparisons of morphological disparity between groups (wild/domestic) for (A) allometry‐corrected and (B) uncorrected shape data (residuals), providing Procrustes Variance values for each group and a P‐value associated with pairwise differences in variances, based on resampling (1000 permutations). Table S6. Results of modularity and integration tests using allometry‐corrected shape data (residuals) (A) and uncorrected shape data (B), using two a priori defined modules for the cranium: the neural crest (NC) and mesoderm (MD). Table S7. Summary of the Principal Component (PC) axes extracted from Principal Component Analysis (PCA) of cranial landmark data for Canis lupus/Canis lupus familiaris, detailing 95% of sample variation. Table S8. Summary of the Principal Component (PC) axes extracted from Principal Component Analysis (PCA) of cranial landmark data for Capra aegagrus/Capra hircus detailing 95% of sample variation. Table S9. Summary of the Principal Component (PC) axes extracted from Principal Component Analysis (PCA) of cranial landmark data for Equus ferus przewalskii/ Equus ferus caballus detailing 95% of sample variation. Table S10. Summary of the Principal Component (PC) axes extracted from Princip [file EVL3-5-385-s002.docx]

**SUPPLEMENTARY DATA FILE**

**Modularity patterns in mammalian domestication: assessing developmental hypotheses for diversification**

Laura A. B. Wilson^1,2^, Ana Balcarcel^3^, Madeleine Geiger^3^, Laura Heck

Marcelo Sanchez-Villagra^3^

**CONTENTS**

**Text S1,** Procrustes Superimposition choice for modularity analyses…………………………………..2

**Text S2,** Module assignment……………………………………………………………………………3

**Text S3,** PLS and PC comparison………………………………………………………………………4

**Table S1**, Description of cranial landmark locations……..………………………………..……………5

**Table S2**, Results of Procrustes ANOVA (shape ~ size) ………..……………………………….…….6

**Table S3**, Pairwise comparisons of vector correlations………………………………………………...7

**Table S4**, T-test comparisons of Centroid size for cranial landmarks………………………………….8

**Table S5**, Multivariate homogeneity of variances test results………………………………………….9

**Table S6**, Results of modularity and integration tests……………………………………………....…10

**Table S7**, Principal Components summary for *Canis lupus/Canis lupus familiaris*…………………...11

**Table S8**, Principal Components summary for *Capra aegagrus/Capra hircus*………………………..12

**Table S9**, Principal Components summary for *Equus ferus przewalskii/Equus ferus caballus*……..…13

**Table S10**, Principal Components summary for *Lama guanicoe/Lama glama*……………..…………14

**Table S11**, Principal Components summary for *Sus scrofa scrofa/Sus scrofa domestica*……………..15

**Table S12**, Principal Components summary for *Vicugna vicugna/Lama pacos*…………………….....16

**Table S13**, Results of vector correlations between Principal Component 1 and Partial Least Squares axis 1………………..…………………………………………………………………………………17

**Table S14**, Effect sizes for pairwise comparisons of Partial Least Squares analyses…………...……18

**Table S15**, Two-sample z-tests for Partial Least Squares analyses…………………………………..19

**Table S16**, Within-module disparity for the Neural Crest and Mesoderm modules………………….20

**Table S17**, Within-module integration magnitudes for the Neural Crest and Mesoderm modules………………………………………………………………………………………….…….21

**Table S18**, Between-module integration magnitudes for the Neural Crest and Mesoderm modules……………………………………………………………………………………..…………22

**Figure S1**, Boxplots of cranial centroid size for wild/domestic pairs………………………………...23

**Figure S2**, Ordinations of shape variation for wild/domestic pairs…………………………………..24

**Text S1: Procrustes superimposition choice for modularity analyses**

The choice of Procrustes superimposition protocol for modularity and integration analyses has been investigated by several authors (Baab 2013; Cardini 2019) and, recently, caution has been raised about how different superimposition approaches may produce different results for the same data set (Cardini 2019). One may perform GPA on landmarks and then subset superimposed landmarks into modules (simultaneous-fit approach, Baab 2013) or one may subset landmarks into modules first, and then perform individual GPA on each module (separate-subset approach, Baab 2013). Our modules represent contiguous skeletal regions (i.e. two parts of the cranium) and our comparison of interest is between modules within domestic/wild pairs, therefore the relative size and position of module 1 vs module 2 is of interest within and between a domestic/wild pair. As such, we follow the recommendation of Baab (2013) to adopt a simultaneous fit approach, followed by subsetting of landmarks into modules.

Baab, K. L. (2013). The impact of superimposition choice in geometric morphometric approaches to morphological integration. J Hum Evol, 65, 689-692.

Cardini, A. (2019). Integration and modularity in Procrustes shape data: is there a risk of spurious results? Evolutionary Biology, 46(1), 90-105.

**Text S2: Module assignment and allometric assessment of module landmarks**

Module assignment was determined for each landmark by the tissue origin of the bone on which a given landmark was located. The origins of cranial bones, as Neural Crest (NC) or Mesoderm (MD) derived, were assigned following Mishina and Snider (2014) for the face and vault elements, and McBratney-Owen et al. (2008) for the cranial base. Landmark #32 was located on the sutural boundary between the NC and MD modules and was assigned to the MD module, as the coronal suture is of mesoderm origin (Mishina and Snider 2014). For the Pig/Wild Boar, Horse/Przewalski’s Horse, Llama/Guanaco and Alpaca/Vicuña pairs, the NC module comprised 47 landmarks, and the MD module comprised 15 landmarks. For the Goat/Bezoar, the NC module contained 45 landmarks, owing to the absence of landmarks 1 and 2. For the Dog/Wolf pair, 19 landmarks were assigned to the NC module, and 7 landmarks were assigned to the MD module.

We assessed our NC-MD modularity hypothesis using the covariance ratio (CR) (Adams 2016) and the modularity.test function in the R package geomorph v.3.1.3 (Adams et al. 2019) for each domestic/wild pair. The CR describes the relationship between the magnitude of covariation between a priori defined modules relative to the covariation within them (Adams 2016). CR ranges from 0 to positive values, where CR <1 indicates that within-module covariation is higher than between-module covariation, supporting a modular structure, and CR >1 reflects greater between-module covariation compared to within-module covariation, supporting an integrated structure. CR was calculated for the landmark data as well as for the allometry-corrected landmark data (residuals) and significance was assessed by random assignment of landmarks to modules, using permutation (1000 replicates) (Adams 2016). Wild and domestic forms were pooled within each pair because modularity patterns have been demonstrated to be stable across placental mammals (Porto et al. 2009; Marroig et al. 2009).

Prior to pooling NC and MD module landmarks for the extraction of residuals, allometric slopes were compared for wild and domestic forms between NC and MD modules. This was done using Procrustes ANOVA implemented with the procD.lm function in geomorph v.3.1.3 (Adams et al. 2019) (i.e. NCshape ~ size * group, MDshape ~ size *group). Fit comparisons were performed using the pairwise function in RRPP v.0.4.3 (Collyer and Adams 2018) and evaluated using the summary.pairwise (“VC”, vector correlation option) function to test the null hypothesis that allometric slopes calculated separately for the NC and MD landmarks were parallel.

Adams, D. C. (2016). Evaluating modularity in morphometric data: challenges with the RV coefficient and a new test measure. *Method Ecol. Evol*. 7(5), 565-572.

Adams, D., Collyer, M., & Kaliontzopoulou, A. (2019). Geomorph: Software for geometric morphometric analyses. R package version 3.1.3.

Collyer, M. L., & Adams, D. C. (2018). RRPP: An r package for fitting linear models to high‐dimensional data using residual randomization. *Methods Ecol. Evol.* 9(7), 1772-1779.

McBratney-Owen, B., Iseki, S., Bamforth, S., Olsen, B., & Morriss-Kay, G. (2008). Development and tissue origins of the mammalian cranial base. *Dev. Biol.* 322(1), 121-132.

Mishina, Y., & Snider, T. N. (2014). Neural crest cell signaling pathways critical to cranial bone development and pathology. *Exp. Cell Res.* 325(2), 138-147.

Marroig, G., Shirai, L. T., Porto, A., de Oliveira, F. B., & De Conto, V. (2009). The Evolution of Modularity in the Mammalian Skull II: Evolutionary Consequences. Evol. Biol. 36(1), 136-148.

Porto, A., de Oliveira, F. B., Shirai, L. T., De Conto, V., & Marroig, G. (2009). The Evolution of Modularity in the Mammalian Skull I: Morphological Integration Patterns and Magnitudes. *Evol. Biol.* 36(1), 118-135.

**Text S3: PLS and PC comparison**

The output of the PLS analysis was used to assess the extent to which the main axis of shape variation in the domestic/wild pairs corresponds with covariation between the NC and MD modules. We extracted the axes explaining most variation in shape space (Principal Component 1, PC1) and most variation between the NC and MD modules (Partial Least Squares 1, PLS1 [or Singular Warp 1]). PC1 was extracted from a Principal Component Analysis (PCA) performed on allometry-corrected landmarks, applied to each of the six domestic/wild pairs separately, using the gm.prcomp function in geomorph v3.1.3 (Adams & Collyer 2016). PLS1 was extracted from the integration.test function results described above, for each domestic/wild pair. Vector correlations were performed between PC1 and PLS1 using Pearson’s product moment correlation with the R base function cor.test, and bivariate plots were examined to assess the relationship for domestic/wild pairs.

Adams, D. C., & Collyer, M. L. (2016). On the comparison of the strength of morphological integration across morphometric datasets. Evolution, 70(11), 2623-2631.

**Table S1.** Description of three dimensional (3D) geometric morphometric landmarks (LM) collected on mammalian crania for wild/domestic pairs in this study and their assignment to neural crest (unshaded) and mesoderm (shaded grey) modules. Landmark data compiled for dog/wolf (see Geiger et al. 2017) represent a subset of the total landmarks collect for the other species and were pruned to include only homologous landmarks (dog/wolf landmarks are indicated with blue coloured font). LM 1-2 was not present in the Goat/Bezoar pair.

| **LM #** | **Description** | **LM #** | **Description** |
| --- | --- | --- | --- |
| 1-2 | Posterior tip of the third upper incisor alvelous (dorsal view) | 36 | Opisthion, dorsal margin of the foramen magnum |
| 3-4 | Anterior-most point of the nasal-premaxilla suture | 37 | Midline between incisive bones, anterior most point (ventral view) |
| 5-6 | Junction of the premaxilla, maxilla, and nasal sutures | 38-39 | Suture between incisive bone and maxilla (lateral view) |
| 7-8 | Dorsoposterior edge of the infraorbital foramen | 40-41 | Anterior edge of P4 alveolus |
| 9-10 | Anterior-most point of the zygomatic arch, in lateral view, inferior edge | 42-43 | Posterior edge of the third molar alveolus |
| 11 | Nasion, nasal-frontal suture, midline | 44 | Posterior-most point of the incisive canal |
| 12-13 | Juncture of the lacrimal, maxilla, and frontal sutures | 45 | Posterior tip of the palatine process of the incisive bone |
| 14-15 | Zygomatic-lacrimal suture on the orbital margin | 46 | Posterior tip of the palatine-palatine suture |
| 16-17 | Lacrimal-frontal suture on the orbital margin | 47-48 | Distal tip of the pterygoid hamulus |
| 18-19 | Supraorbital foramen | 49-50 | Anterior edge of the caudal alar foramen |
| 20-21 | Anterior tip of the temporal-zygomatic suture | 51 | Posterior tip of the vomer on the midline |
| 22-23 | Posterior tip of the temporal-zygomatic suture | 52-53 | Small fossa medial to mandibular fossa and retroarticular process |
| 24-25 | Fronto-zygomatic suture on posterior orbit, midpoint | 54-55 | Hypoglossal nerve canal, anterior margin |
| 26-27 | Articular eminence apex | 56-57 | Fossa medial of the paracondylar process (central point) |
| 28-29 | Dorsal-most point of the vertically oriented posterior margin of the zygomatic process | 58-59 | Distal tip of the paracondylar process |
| 30-31 | Posterior-most point of the vertically oriented posterior margin of the zygomatic process | 60 | Basion, midpoint on ventral margin of foramen magnum |
| 32 | Bregma, intersection of interfrontal and interparietal suture | 61-62 | Posterior-most tips of the occipital condyles |
| 33 | Inion, highest projection of the external occipital protuberance (dorsal) |  |  |
| 34-35 | Dorsolateral-most edge of the nuchal crest |  |  |

**Table S2.** Results of Procrustes ANOVA (shape ~ size), test statistics based on Residual Randomization (1000 permutations).

| Wild/domestic pair | SS | MS | Rsq | F | Z | P |
| --- | --- | --- | --- | --- | --- | --- |
| *Canis lupus*  *Canis lupus familiaris* | 0.038857 | 0.038857 | 0.14957 | 12.9965 | 4.8970 | **0.001** |
| *Capra aegagrus*  *Capra hircus* | 0.03129 | 0.031294 | 0.06449 | 4.5718 | 3.6249 | **0.001** |
| *Equus ferus przewalskii Equus ferus caballus* | 0.05747 | 0.057469 | 0.11570 | 34.076 | 8.5276 | **0.001** |
| *Lama guanicoe*  *Lama glama* | 0.016794 | 0.0167940 | 0.06020 | 6.8062 | 5.9060 | **0.001** |
| *Sus scrofa scrofa*  *Sus scrofa domestica* | 0.14109 | 0.141093 | 0.23265 | 27.7865 | 3.2138 | **0.001** |
| *Vicugna vicugna*  *Lama pacos* | 0.011201 | 0.0112009 | 0.10559 | 4.2332 | 3.8876 | **0.001** |

**Table S3.** Pairwise statistics for comparisons of LS mean vector correlations between wild/domestic forms and between landmarks in the Neural Crest (NC) and Mesoderm (MD) modules based on residual randomization 1000 permutations. The null hypothesis is that the angle between vectors = 0.

| **A. Wild/domestic comparison** | | | |
| --- | --- | --- | --- |
| Wild/domestic pair | Angle between vectors (degrees) | Upper Confidence Limit (UCL) 95% | P |
| *Canis lupus*  *Canis lupus familiaris* | 2.460 | 3.239 | 0.924 |
| *Capra aegagrus*  *Capra hircus* | 2.975 | 3.650 | 0.885 |
| *Equus ferus przewalskii Equus ferus caballus* | 2.146 | 2.281 | 0.647 |
| *Lama guanicoe*  *Lama glama* | 1.425 | 2.166 | 0.931 |
| *Sus scrofa scrofa*  *Sus scrofa domestica* | 8.917 | 10.323 | 0.533 |
| *Vicugna vicugna*  *Lama pacos* | 2.549 | 3.283 | 0.644 |
| **B. NC and MD partitions** | |  |  |
| Wild/domestic pair | Angle between vectors (degrees) | Upper Confidence Limit (UCL) 95% | P |
| *Canis lupus*  *Canis lupus familiaris* | 110.278 | 131.631 | 0.33 |
| *Capra aegagrus*  *Capra hircus* | 91.104 | 113.563 | 0.485 |
| *Equus ferus przewalskii Equus ferus caballus* | 102.146 | 125.029 | 0.471 |
| *Lama guanicoe*  *Lama glama* | 76.565 | 101.925 | 0.639 |
| *Sus scrofa scrofa*  *Sus scrofa domestica* | 142.96 | 146.341 | 0.12 |
| *Vicugna vicugna*  *Lama pacos* | 76.565 | 101.925 | 0.639 |

**Table S4.** Welch’s two sample T-test results for comparisons of log centroid size between wild and domestic forms. Centroid size was calculated for each individual using cranial landmarks, as per Table S1. Boxplots of these data are also provided in Figure S1.

| Wild/domestic pair | Mean Centroid size  domestic | Mean Centroid size  wild | t | P |
| --- | --- | --- | --- | --- |
| *Canis lupus*  *Canis lupus familiaris* | 5.847 | 6.032 | -8.268 | **9.432e-12** |
| *Capra aegagrus*  *Capra hircus* | 6.296 | 6.282 | 0.445 | 0.658 |
| *Equus ferus przewalskii Equus ferus caballus* | 7.173 | 7.094 | 4.916 | **1.87e-06** |
| *Lama guanicoe*  *Lama glama* | 6.530 | 6.554 | -2.264 | **0.0317** |
| *Sus scrofa scrofa*  *Sus scrofa domestica* | 6.667 | 6.782 | -3.606 | **0.0011** |
| *Vicugna vicugna*  *Lama pacos* | 6.414 | 6.304 | 4.776 | **5.979e-05** |
|  |  |  |  |  |

**Table S5.** Comparisons of morphological disparity between groups (wild/domestic) for (A) allometry-corrected and (B) uncorrected shape data (residuals), providing Procrustes Variance values for each group and a P-value associated with pairwise differences in variances, based on resampling (1000 permutations).

| **A. Allometry-corrected**  Wild/domestic pair | Procrustes Variance domestic | Procrustes Variance wild | P | Greatest Variance (group) |
| --- | --- | --- | --- | --- |
| *Canis lupus*  *Canis lupus familiaris* | 0.0038 | 0.0011 | **0.001** | domestic |
| *Capra aegagrus*  *Capra hircus* | 0.0082 | 0.0031 | **0.019** | domestic |
| *Equus ferus przewalskii Equus ferus caballus* | 0.0015 | 0.0019 | **0.001** | wild |
| *Lama guanicoe*  *Lama glama* | 0.0026 | 0.0023 | 0.385 | domestic |
| *Sus scrofa scrofa*  *Sus scrofa domestica* | 0.0083 | 0.0018 | **0.001** | domestic |
| *Vicugna vicugna*  *Lama pacos* | 0.0026 | 0.0022 | 0.217 | domestic |
| **B. Uncorrected shape data**  Wild/domestic pair | Procrustes Variance domestic | Procrustes Variance wild | P | Greatest Variance (group) |
| *Canis lupus*  *Canis lupus familiaris* | 0.0046 | 0.0022 | **0.001** | domestic |
| *Capra aegagrus*  *Capra hircus* | 0.0093 | 0.0047 | **0.041** | domestic |
| *Equus ferus przewalskii Equus ferus caballus* | 0.0020 | 0.0028 | **0.001** | wild |
| *Lama guanicoe*  *Lama glama* | 0.0036 | 0.0025 | **0.012** | domestic |
| *Sus scrofa scrofa*  *Sus scrofa domestica* | 0.0180 | 0.0075 | **0.001** | domestic |
| *Vicugna vicugna*  *Lama pacos* | 0.0037 | 0.0026 | **0.006** | domestic |

**Table S6.** Results of modularity and integration tests using allometry-corrected shape data (residuals) (A) and uncorrected shape data (B), using two a priori defined modules for the cranium: the neural crest (NC) and mesoderm (MD). Modularity is quantified using Covariance Ratio (CR), which ranges between 0 to positive values, whereby CR <1 indicates that within-module covariation is higher than between-module covariation, supporting a modular structure, and CR >1 reflects greater between-module covariation compared to within-module covariation, supporting an integrated structure. The magnitude of integration between the NC and MD modules was quantified using the mean pairwise partial least squares correlations (r-PLS).

| **A – Allometry corrected** | Modularity |  | Integration |  |
| --- | --- | --- | --- | --- |
| Wild/domestic pair | CR | P | r-PLS | P |
| *Canis lupus*  *Canis lupus familiaris* | 0.7694 | **0.002** | 0.765 | **0.001** |
| *Capra aegagrus*  *Capra hircus* | 0.8513 | 0.205 | 0.8 | **0.001** |
| *Equus ferus przewalskii Equus ferus caballus* | 0.7676 | **0.001** | 0.883 | **0.001** |
| *Lama guanicoe*  *Lama glama* | 0.7731 | **0.002** | 0.815 | **0.001** |
| *Sus scrofa scrofa*  *Sus scrofa domestica* | 0.8865 | **0.001** | 0.883 | **0.001** |
| *Vicugna vicugna*  *Lama pacos* | 0.7853 | **0.001** | 0.906 | **0.001** |
|  |  |  |  |  |
|  |  |  |  |  |
| **B – Uncorrected shape data** | Modularity |  | Integration |  |
| Wild/domestic pair | CR | P | r-PLS | P |
| *Canis lupus*  *Canis lupus familiaris* | 0.8102 | **0.003** | 0.799 | **0.001** |
| *Capra aegagrus*  *Capra hircus* | 0.7999 | **0.045** | 0.772 | **0.001** |
| *Equus ferus przewalskii Equus ferus caballus* | 0.7638 | **0.001** | 0.87 | **0.001** |
| *Lama guanicoe*  *Lama glama* | 0.8027 | **0.004** | 0.844 | **0.001** |
| *Sus scrofa scrofa*  *Sus scrofa domestica* | 0.9685 | **0.001** | 0.948 | **0.001** |
| *Vicugna vicugna*  *Lama pacos* | 0.8282 | **0.002** | 0.909 | **0.001** |
|  |  |  |  |  |

**Table S7.** Summary of the Principal Component (PC) axes extracted from Principal Component Analysis (PCA) of cranial landmark data for *Canis lupus*/*Canis lupus familiaris*, detailing 95% of sample variation.

|  | **PC1** | **PC2** | **PC3** | **PC4** | **PC5** | **PC6** | **PC7** | **PC8** | **PC9** |
| --- | --- | --- | --- | --- | --- | --- | --- | --- | --- |
| Standard deviation | 0.03573 | 0.02419 | 0.01848 | 0.01691 | 0.01395 | 0.01213 | 0.01087 | 0.009546 | 0.009279 |
| Proportion of Variance | 0.33751 | 0.15466 | 0.09025 | 0.07556 | 0.05142 | 0.03892 | 0.03125 | 0.024090 | 0.022760 |
| Cumulative Proportion | 0.33751 | 0.4921 | 0.58242 | 0.65798 | 0.70940 | 0.74832 | 0.77957 | 0.803660 | 0.826420 |
|  | **PC10** | **PC11** | **PC12** | **PC13** | **PC14** | **PC15** | **PC16** | **PC17** | **PC18** |
| Standard deviation | 0.008705 | 0.007957 | 0.007749 | 0000678 | 0.006434 | 0.006129 | 0.005834 | 0.005653 | 0.005536 |
| Proportion of Variance | 0.020030 | 0.016740 | 0.015870 | 0.01215 | 0.010950 | 0.009930 | 0.00900 | 0.008450 | 0.008100 |
| Cumulative Proportion | 0.826420 | 0.863190 | 0.879070 | 0.89122 | 0.902160 | 0.912090 | 0.921090 | 0.929540 | 0.937640 |
|  | **PC19** | **PC20** |  |  |  |  |  |  |  |
| Standard deviation | 0.004963 | 0.004834 |  |  |  |  |  |  |  |
| Proportion of Variance | 0.006510 | 0.006180 |  |  |  |  |  |  |  |
| Cumulative Proportion | 0.944150 | 0.955580 |  |  |  |  |  |  |  |

**Table S8.** Summary of the Principal Component (PC) axes extracted from Principal Component Analysis (PCA) of cranial landmark data for *Capra aegagrus/Capra hircus* detailing 95% of sample variation.

|  | **PC1** | **PC2** | **PC3** | **PC4** | **PC5** | **PC6** | **PC7** | **PC8** | **PC9** |
| --- | --- | --- | --- | --- | --- | --- | --- | --- | --- |
| Standard deviation | 0.04523 | 0.03487 | 0.02821 | 0.02565 | 0.01998 | 0.01935 | 0.01763 | 0.01499 | 0.01420 |
| Proportion of Variance | 0.25656 | 0.15252 | 0.09979 | 0.08250 | 0.05009 | 0.04697 | 0.03899 | 0.02820 | 0.02531 |
| Cumulative Proportion | 0.25656 | 0.40908 | 0.50887 | 0.59137 | 0.64146 | 0.68842 | 0.72741 | 0.75561 | 0.78091 |
|  | **PC10** | **PC11** | **PC12** | **PC13** | **PC14** | **PC15** | **PC16** | **PC17** | **PC18** |
| Standard deviation | 0.01366 | 0.01224 | 0.01137 | 0.01040 | 0.009759 | 0.009474 | 0.009307 | 0.008967 | 0.008885 |
| Proportion of Variance | 0.02341 | 0.01879 | 0.01622 | 0.01356 | 0.011940 | 0.011260 | 0.010870 | 0.010090 | 0.009900 |
| Cumulative Proportion | 0.80432 | 0.82311 | 0.83933 | 0.85290 | 0.864840 | 0.876100 | 0.886970 | 0.897050 | 0.906950 |
|  | **PC19** | **PC20** | **PC21** | **PC22** | **PC23** | **PC24** | **PC25** | **PC26** |  |
| Standard deviation | 0.007775 | 0.007517 | 0.007284 | 0.006817 | 0.006716 | 0.006192 | 0.005983 | 0.00590 |  |
| Proportion of Variance | 0.007580 | 0.007090 | 0.006650 | 0.005830 | 0.005660 | 0.004810 | 0.004490 | 0.00437 |  |
| Cumulative Proportion | 0.914540 | 0.921620 | 0.928280 | 0.934110 | 0.939760 | 0.944570 | 0.949060 | 0.95343 |  |

**Table S9.** Summary of the Principal Component (PC) axes extracted from Principal Component Analysis (PCA) of cranial landmark data for *Equus ferus przewalskii/ Equus ferus caballus* detailing 95% of sample variation.

|  | **PC1** | **PC2** | **PC3** | **PC4** | **PC5** | **PC6** | **PC7** | **PC8** | **PC9** |
| --- | --- | --- | --- | --- | --- | --- | --- | --- | --- |
| Standard deviation | 0.02021 | 0.01878 | 0.01557 | 0.01248 | 0.01112 | 0.01006 | 0.008175 | 0.007612 | 0.007576 |
| Proportion of Variance | 0.17636 | 0.15226 | 0.10472 | 0.06724 | 0.05341 | 0.04365 | 0.028860 | 0.025020 | 0.024780 |
| Cumulative Proportion | 0.17636 | 0.32863 | 0.43335 | 0.50058 | 0.55399 | 0.59765 | 0.626500 | 0.651520 | 0.676300 |
|  | **PC10** | **PC11** | **PC12** | **PC13** | **PC14** | **PC15** | **PC16** | **PC17** | **PC18** |
| Standard deviation | 0.006857 | 0.006591 | 0.006148 | 0.00609 | 0.005722 | 0.00564 | 0.005485 | 0.05207 | 0.004983 |
| Proportion of Variance | 0.020300 | 0.018750 | 0.016320 | 0.01601 | 0.014140 | 0.01373 | 0.012990 | 0.011700 | 0.010720 |
| Cumulative Proportion | 0.696590 | 0.715340 | 0.731660 | 0.74767 | 0.761810 | 0.77554 | 0.788530 | 0.800230 | 0.810950 |
|  | **PC19** | **PC20** | **PC21** | **PC22** | **PC23** | **PC24** | **PC25** | **PC26** | **PC27** |
| Standard deviation | 0.004905 | 0.004728 | 0.004503 | 0.004393 | 0.004308 | 0.004208 | 0.004098 | 0.003885 | 0.003784 |
| Proportion of Variance | 0.010390 | 0.009650 | 0.008750 | 0.008330 | 0.008010 | 0.007640 | 0.007250 | 0.006520 | 0.006180 |
| Cumulative Proportion | 0.821340 | 0.830990 | 0.839740 | 0.848070 | 0.856090 | 0.863730 | 0.870980 | 0.877500 | 0.883680 |
|  | **PC28** | **PC29** | **PC30** | **PC31** | **PC32** | **PC33** | **PC34** | **PC35** | **PC36** |
| Standard deviation | 0.003681 | 0.003603 | 0.003469 | 0.003384 | 0.003252 | 0.00314 | 0.003108 | 0.00303 | 0.003019 |
| Proportion of Variance | 0.005850 | 0.005610 | 0.005200 | 0.004940 | 0.004570 | 0.00426 | 0.004170 | 0.00396 | 0.003930 |
| Cumulative Proportion | 0.889530 | 0.895130 | 0.900330 | 0.905280 | 0.909840 | 0.91410 | 0.918270 | 0.92223 | 0.926160 |
|  | **PC37** | **PC38** | **PC39** | **PC40** | **PC41** | **PC42** | **PC43** | **PC44** |  |
| Standard deviation | 0.002989 | 0.002938 | 0.002838 | 0.02758 | 0.002665 | 0.002597 | 0.002534 | 0.002402 |  |
| Proportion of Variance | 0.003860 | 0.003730 | 0.003480 | 0.003280 | 0.003070 | 0.002910 | 0.002770 | 0.002490 |  |
| Cumulative Proportion | 0.930020 | 0.933750 | 0.937230 | 0.940510 | 0.943580 | 0.946490 | 0.949260 | 0.951750 |  |
|  |  |  |  |  |  |  |  |  |  |
|  |  |  |  |  |  |  |  |  |  |

**Table S10.** Summary of the Principal Component (PC) axes extracted from Principal Component Analysis (PCA) of cranial landmark data for *Lama guanicoe/Lama glama* detailing 95% of sample variation.

|  | **PC1** | **PC2** | **PC3** | **PC4** | **PC5** | **PC6** | **PC7** | **PC8** | **PC9** |
| --- | --- | --- | --- | --- | --- | --- | --- | --- | --- |
| Standard deviation | 0.02177 | 0.01748 | 0.01442 | 0.01314 | 0.01272 | 0.01064 | 0.01012 | 0.009799 | 0.009122 |
| Proportion of Variance | 0.17429 | 0.11239 | 0.07647 | 0.06352 | 0.05954 | 0.04162 | 0.03764 | 0.035320 | 0.030610 |
| Cumulative Proportion | 0.17429 | 0.28669 | 0.36316 | 0.42667 | 0.48621 | 0.52783 | 0.56547 | 0.600790 | 0.631400 |
|  | **PC10** | **PC11** | **PC12** | **PC13** | **PC14** | **PC15** | **PC16** | **PC17** | **PC18** |
| Standard deviation | 0.008597 | 0.008188 | 0.00776 | 0.007502 | 0.007217 | 0.007062 | 0.006547 | 0.006378 | 0.006065 |
| Proportion of Variance | 0.027190 | 0.024660 | 0.02215 | 0.020700 | 0.019160 | 0.018340 | 0.015770 | 0.014960 | 0.013530 |
| Cumulative Proportion | 0.658580 | 0.683240 | 0.70539 | 0.726100 | 0.745260 | 0.763600 | 0.779370 | 0.794330 | 0.807860 |
|  | **PC19** | **PC20** | **PC21** | **PC22** | **PC23** | **PC24** | **PC25** | **PC26** | **PC27** |
| Standard deviation | 0.005906 | 0.005742 | 0.005549 | 0.005054 | 0.004916 | 0.004869 | 0.004814 | 0.004628 | 0.004503 |
| Proportion of Variance | 0.012830 | 0.012130 | 0.011330 | 0.009400 | 0.008890 | 0.008720 | 0.008520 | 0.007880 | 0.007460 |
| Cumulative Proportion | 0.820690 | 0.832820 | 0.844140 | 0.853540 | 0.862430 | 0.871150 | 0.879680 | 0.887550 | 0.895010 |
|  | **PC28** | **PC29** | **PC30** | **PC31** | **PC32** | **PC33** | **PC34** | **PC35** | **PC36** |
| Standard deviation | 0.004343 | 0.004217 | 0.003957 | 0.003885 | 0.003765 | 0.003722 | 0.003616 | 0.003403 | 0.003262 |
| Proportion of Variance | 0.006940 | 0.006540 | 0.005760 | 0.005550 | 0.005220 | 0.005090 | 0.004810 | 0.004260 | 0.003910 |
| Cumulative Proportion | 0.901950 | 0.908490 | 0.914250 | 0.919800 | 0.925020 | 0.930110 | 0.934920 | 0.939180 | 0.943090 |
|  | **PC37** | **PC38** |  |  |  |  |  |  |  |
| Standard deviation | 0.003185 | 0.00309 |  |  |  |  |  |  |  |
| Proportion of Variance | 0.003730 | 0.00351 |  |  |  |  |  |  |  |
| Cumulative Proportion | 0.946820 | 0.95034 |  |  |  |  |  |  |  |
|  |  |  |  |  |  |  |  |  |  |
|  |  |  |  |  |  |  |  |  |  |

**Table S11.** Summary of the Principal Component (PC) axes extracted from Principal Component Analysis (PCA) of cranial landmark data for *Sus scrofa scrofa/Sus scrofa domestica* detailing 95% of sample variation.

|  | **PC1** | **PC2** | **PC3** | **PC4** | **PC5** | **PC6** | **PC7** | **PC8** | **PC9** |
| --- | --- | --- | --- | --- | --- | --- | --- | --- | --- |
| Standard deviation | 0.09855 | 0.02698 | 0.01971 | 0.01665 | 0.01432 | 0.01300 | 0.01131 | 0.01089 | 0.009935 |
| Proportion of Variance | 0.76176 | 0.05709 | 0.03047 | 0.02173 | 0.01609 | 0.01326 | 0.01004 | 0.00930 | 0.007740 |
| Cumulative Proportion | 0.76176 | 0.81885 | 0.84932 | 0.87105 | 0.88714 | 0.90040 | 0.91044 | 0.91974 | 0.927480 |
|  | **PC10** | **PC11** | **PC12** | **PC13** |  |  |  |  |  |
| Standard deviation | 0.009545 | 0.009161 | 0.00858 | 0.008375 |  |  |  |  |  |
| Proportion of Variance | 0.007740 | 0.006580 | 0.00577 | 0.005500 |  |  |  |  |  |
| Cumulative Proportion | 0.927480 | 0.941210 | 0.94699 | 0.952490 |  |  |  |  |  |

**Table S12.** Summary of the Principal Component (PC) axes extracted from Principal Component Analysis (PCA) of cranial landmark data for *Vicugna vicugna/Lama pacos* detailing 95% of sample variation.

|  | **PC1** | **PC2** | **PC3** | **PC4** | **PC5** | **PC6** | **PC7** | **PC8** | **PC9** |
| --- | --- | --- | --- | --- | --- | --- | --- | --- | --- |
| Standard deviation | 0.02421 | 0.02175 | 0.01640 | 0.01596 | 0.01542 | 0.01278 | 0.01106 | 0.01058 | 0.01004 |
| Proportion of Variance | 0.18719 | 0.15114 | 0.08591 | 0.08133 | 0.07590 | 0.05218 | 0.03904 | 0.03577 | 0.026940 |
| Cumulative Proportion | 0.18719 | 0.33833 | 0.42423 | 0.50557 | 0.58147 | 0.63365 | 0.67269 | 0.70846 | 0.767590 |
|  | **PC10** | **PC11** | **PC12** | **PC13** | **PC14** | **PC15** | **PC16** | **PC17** | **PC18** |
| Standard deviation | 0.009184 | 0.008733 | 0.00844 | 0.007735 | 0.007618 | 0.007288 | 0.006857 | 0.006767 | 0.006196 |
| Proportion of Variance | 0.026940 | 0.024360 | 0.02275 | 0.019110 | 0.018530 | 0.016960 | 0.015020 | 0.014630 | 0.012260 |
| Cumulative Proportion | 0.767590 | 0.791950 | 0.81470 | 0.833810 | 0.852340 | 0.869300 | 0.884320 | 0.898950 | 0.911210 |
|  | **PC19** | **PC20** | **PC21** | **PC22** |  |  |  |  |  |
| Standard deviation | 0.006115 | 0.005787 | 0.00539 | 0.00512 |  |  |  |  |  |
| Proportion of Variance | 0.011940 | 0.010700 | 0.00928 | 0.00837 |  |  |  |  |  |
| Cumulative Proportion | 0.923150 | 0.933850 | 0.94313 | 0.95150 |  |  |  |  |  |

**Table S13.** Results of vector correlations between Principal Component 1 (PC1) and Partial Least Squares axis 1 (PLS1) for landmark data and allometry-corrected landmark data.

|  | **Allometry-corrected data (residuals)** | | **Uncorrected shape data** | |
| --- | --- | --- | --- | --- |
|  | **Correlation** | **P** | **Correlation** | **P** |
| *Canis lupus/*  *Canis lupus familiaris* | -0.6282 | **5.82e-09** | -0.7915 | **3.483e-16** |
| *Capra aegagrus/*  *Capra hircus* | 0.4038 | **0.0010** | 0.4208 | **0.00059** |
| *Equus ferus przewalskii/*  *Equus ferus caballus* | 0.3926 | **2.259e-09** | -0.7262 | **2.2e-16** |
| *Lama guanicoe/*  *Lama glama* | 0.0593 | 0.5498 | 0.0792 | 0.4238 |
| *Sus scrofa scrofa/*  *Sus scrofa domestica* | -0.4741 | **0.0005** | -0.9692 | **2.2e-16** |
| *Vicugna vicugna/*  *Lama pacos* | 0.0320 | 0.8554 | -0.3090 | 0.07088 |

**Table S14.** Effect sizes (z) extracted from pairwise comparisons of Partial Least Squares (PLS) analyses for landmark data and allometry-corrected landmark data.

| Wwild/domestic pair | Allometry-corrected  z | Uncorrected shape data  z |
| --- | --- | --- |
| *Capra aegagrus/*  *Capra hircus* | 4.9953 | 4.9330 |
| *Equus ferus przewalskii/*  *Equus ferus caballus* | 13.783 | 15.835 |
| *Lama guanicoe/*  *Lama glama* | 7.3479 | 8.4665 |
| *Sus scrofa scrofa/*  *Sus scrofa domestica* | 7.6071 | 9.7688 |
| *Vicugna vicugna/*  *Lama pacos* | 4.0925 | 4.8047 |

**Table S15.** Pairwise comparison of effect size (z) (raw values presented in Table S12) using two-sample z-tests of PLS analyses, showing P values for allometry-corrected data (below diagonal) and uncorrected data (above diagonal).

|  | *Capra aegagrus/*  *Capra hircus* | *Equus ferus przewalskii/*  *Equus ferus caballus* | *Lama guanicoe/*  *Lama glama* | *Sus scrofa scrofa/*  *Sus scrofa domestica* | *Vicugna vicugna/*  *Lama pacos* |
| --- | --- | --- | --- | --- | --- |
| *Capra aegagrus/*  *Capra hircus* |  | **1.234e-14** | **0.0124** | **6.236e-04** | 0.0928 |
| *Equus ferus przewalskii/*  *Equus ferus caballus* | **5.058e-10** |  | **1.862e-07** | **1.0e-05** | **6.036e-15** |
| *Lama guanicoe/*  *Lama glama* | 0.0960 | **5.292e-06** |  | 0.3569 | **9.582e-03** |
| *Sus scrofa scrofa/*  *Sus scrofa domestica* | 0.0643 | **1.245e-05** | 0.0855 |  | **0.00044** |
| *Vicugna vicugna/*  *Lama pacos* | 0.5230 | **7.082e-12** | **0.0213** | **0.0129** |  |

**Table S16.** Morphological disparity values, measuring within-module disparity for the Neural Crest (NC) and Mesoderm (MD) modules, for wild and domestic forms. Values calculated for A) Allometry-corrected and B) Uncorrected shape data. All disparity values corrected for unequal number of landmarks within each module (disparity/N landmarks).

| **A – Allometry corrected** | | | **Disparity (MD)** | **P** | **Disparity (NC)** | **P** |
| --- | --- | --- | --- | --- | --- | --- |
|  | | |  |  |  |  |
| wild | *Canis lupus* | | 2.545E-05 | **0.001** | 3.387E-05 | **0.001** |
| domestic | *Canis lupus familiaris* | | 1.139E-04 |  | 1.137E-04 |  |
| wild | *Capra aegagrus* | | 5.058E-05 | **0.13** | 5.165E-05 | **0.005** |
| domestic | *Capra hircus* | | 1.745E-04 |  | 1.240E-04 |  |
| wild | *Equus ferus przewalskii* | | 2.535E-05 | **0.009** | 2.363E-05 | **0.001** |
| domestic | *Equus ferus caballus* | | 3.099E-05 |  | 3.094E-05 |  |
| wild | *Lama guanicoe* | | 2.923E-05 | 0.701 | 3.994E-05 | 0.402 |
| domestic | *Lama glama* | | 3.149E-05 |  | 4.623E-05 |  |
| wild | *Sus scrofa scrofa* | | 3.166E-05 | **0.001** | 2.899E-05 | **0.001** |
| domestic | *Sus scrofa domestica* | | 1.477E-04 |  | 1.290E-04 |  |
| wild | *Vicugna vicugna* | | 2.406E-05 | **0.018** | 3.894E-05 | 0.408 |
| domestic | *Lama pacos* | | 3.318E-05 |  | 4.414E-05 |  |
|  |  | |  |  |  |  |
| **B – Uncorrected shape data** | | | **Disparity (MD)** | **P** | **Disparity (NC)** | **P** |
| wild | | *Canis lupus* | 5.271E-05 | **0.001** | 6.630E-05 | **0.001** |
| domestic | | *Canis lupus familiaris* | 1.305E-04 |  | 1.365E-04 |  |
| wild | | *Capra aegagrus* | 7.283E-05 | 0.178 | 8.073E-05 | **0.007** |
| domestic | | *Capra hircus* | 1.893E-04 |  | 1.442E-04 |  |
| wild | | *Equus ferus przewalskii* | 4.522E-05 | **0.001** | 4.522E-05 | **0.001** |
| domestic | | *Equus ferus caballus* | 3.209E-05 |  | 3.199E-05 |  |
| wild | | *Lama guanicoe* | 3.080E-05 | 0.059 | 4.319E-05 | **0.014** |
| domestic | | *Lama glama* | 4.143E-05 |  | 6.318E-05 |  |
| wild | | *Sus scrofa scrofa* | 1.609E-04 | **0.001** | 1.082E-04 | **0.002** |
| domestic | | *Sus scrofa domestica* | 3.639E-03 |  | 2.672E-04 |  |
| wild | | *Vicugna vicugna* | 3.053E-05 | **0.003** | 4.518E-05 | **0.017** |
| domestic | | *Lama pacos* | 4.966E-05 |  | 6.298E-05 |  |

**Table S17.** Eigenvalue dispersion values, measuring integration magnitude within the Neural Crest (NC) and Mesoderm (MD) modules, for wild and domestic forms. Integration values are corrected for unequal number of landmarks within each module (relative standard deviation, see Pavlicev et al. 2009).

|  |  | **Integration within MD** | **Integration within NC** |
| --- | --- | --- | --- |
| wild | *Canis lupus* | 0.0529 | 0.0117 |
| domestic | *Canis lupus familiaris* | 0.0648 | 0.0164 |
| wild | *Capra aegagrus* | 0.0242 | 0.0057 |
| domestic | *Capra hircus* | 0.0215 | 0.0056 |
| wild | *Equus ferus przewalskii* | 0.0215 | 0.0053 |
| domestic | *Equus ferus caballus* | 0.0211 | 0.0049 |
| wild | *Lama guanicoe* | 0.0185 | 0.0044 |
| domestic | *Lama glama* | 0.0175 | 0.0060 |
| wild | *Sus scrofa scrofa* | 0.0239 | 0.0050 |
| domestic | *Sus scrofa domestica* | 0.0351 | 0.0084 |
| wild | *Vicugna vicugna* | 0.0156 | 0.0051 |
| domestic | *Lama pacos* | 0.0171 | 0.0058 |
|  |  |  |  |
| Average (pooled) |  | 0.0278 | 0.007 |
| Average (wild) |  | 0.0261 | 0.006 |
| Average (domestic) |  | 0.0295 | 0.008 |

**Table S18.** Between-module integration values for wild and domestic forms, assessing integration between the Neural Crest (NC) and Mesoderm (MD) modules.

|  |  | **Integration between NC-MD** | **Allometry-corrected Integration between NC-MD** |
| --- | --- | --- | --- |
| wild | *Canis lupus* | 0.789 | 0.837 |
| domestic | *Canis lupus familiaris* | 0.795 | 0.789 |
| wild | *Capra aegagrus* | 0.933 | 0.931 |
| domestic | *Capra hircus* | 0.8 | 0.805 |
| wild | *Equus ferus przewalskii* | 0.807 | 0.892 |
| domestic | *Equus ferus caballus* | 0.86 | 0.884 |
| wild | *Lama guanicoe* | 0.803 | 0.796 |
| domestic | *Lama glama* | 0.889 | 0.901 |
| wild | *Sus scrofa scrofa* | 0.766 | 0.769 |
| domestic | *Sus scrofa domestica* | 0.913 | 0.925 |
| wild | *Vicugna vicugna* | 0.921 | 0.923 |
| domestic | *Lama pacos* | 0.863 | 0.801 |
|  |  |  |  |
| Average (pooled) |  | 0.845 | 0.854 |
| Average (wild) |  | 0.853 | 0.851 |
| Average (domestic) |  | 0.837 | 0.858 |

**Figure S1.** Boxplots of log cranial centroid size for wild (yellow) and domestic (blue) mammal pairs. Centroid size was calculated for each specimen using cranial landmark data. These vectors were compared using a t-test (see Table S4 for corresponding results).

**
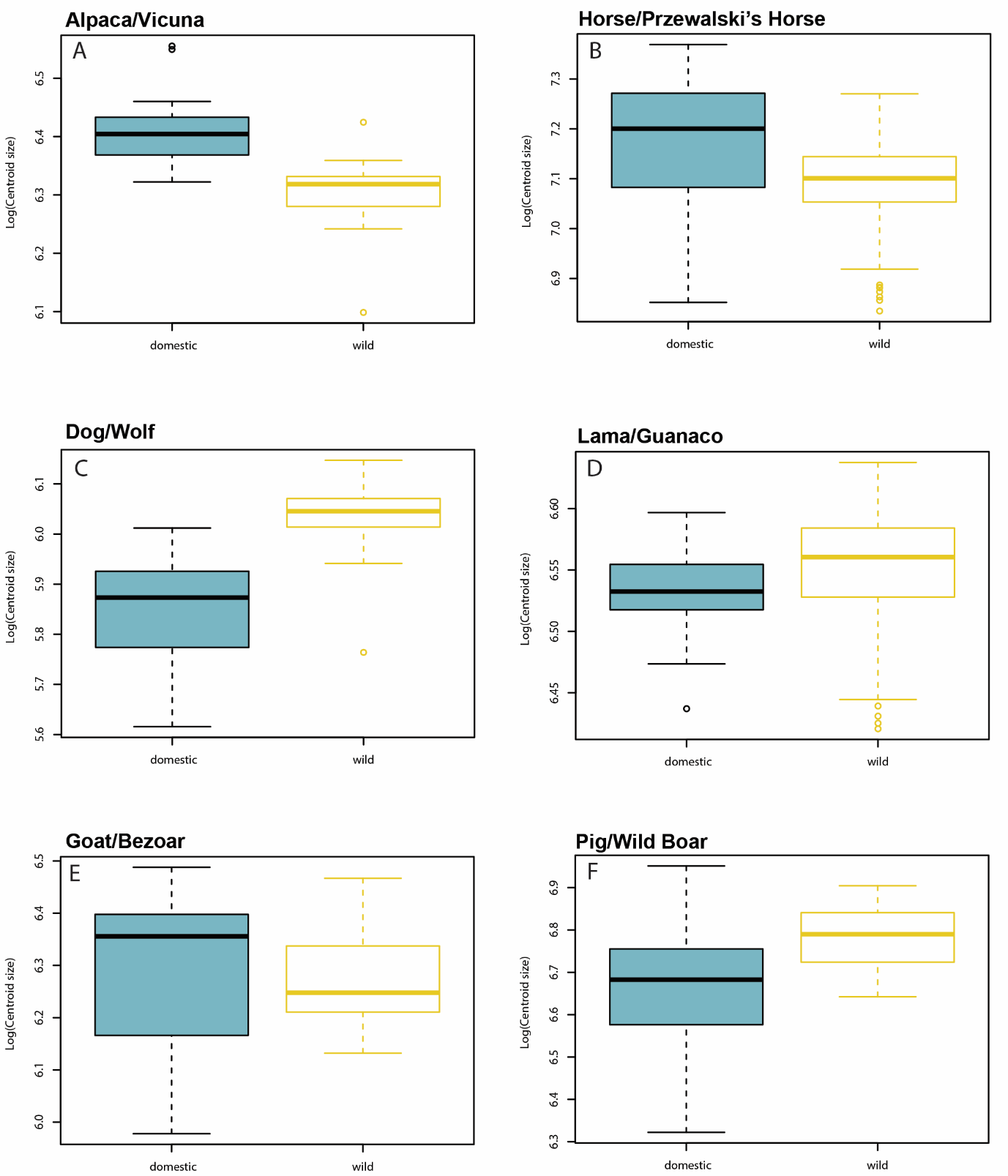
**

**Figure S2.** Ordinations of uncorrected landmark data, for wild (yellow) and domestic (blue) forms. Morphospaces show Principal Coordinate Axes (PCoA) 1 and 2 and illustrate Euclidean distances between individuals and the group centroid. For all comparisons except Horse/Przewalski’s Horse, variance among domestic forms was greater than that among wild forms. See Table S5.

**
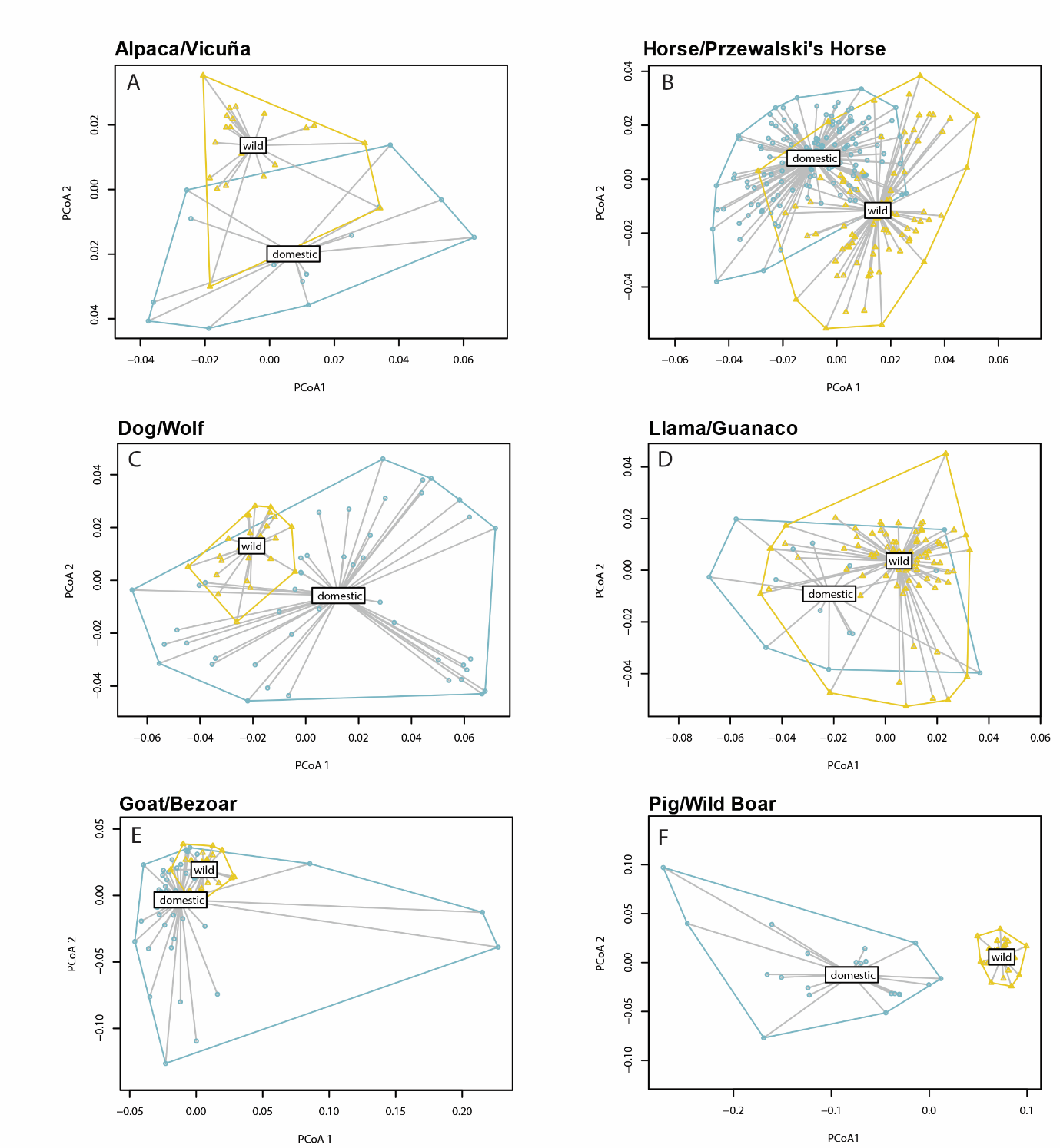
**
